# Supervised Learning for Detection of Duplicates in Genomic Sequence Databases

**DOI:** 10.1371/journal.pone.0159644

**Published:** 2016-08-04

**Authors:** Qingyu Chen, Justin Zobel, Xiuzhen Zhang, Karin Verspoor

**Affiliations:** 1 Department of Computing and Information Systems, The University of Melbourne, Melbourne, Australia; 2 School of Science, RMIT University, Melbourne, Australia; University of Lausanne, SWITZERLAND

## Abstract

**Motivation:**

First identified as an issue in 1996, duplication in biological databases introduces redundancy and even leads to inconsistency when contradictory information appears. The amount of data makes purely manual de-duplication impractical, and existing automatic systems cannot detect duplicates as precisely as can experts. Supervised learning has the potential to address such problems by building automatic systems that learn from expert curation to detect duplicates precisely and efficiently. While machine learning is a mature approach in other duplicate detection contexts, it has seen only preliminary application in genomic sequence databases.

**Results:**

We developed and evaluated a supervised duplicate detection method based on an expert curated dataset of duplicates, containing over one million pairs across five organisms derived from genomic sequence databases. We selected 22 features to represent distinct attributes of the database records, and developed a binary model and a multi-class model. Both models achieve promising performance; under cross-validation, the binary model had over 90% accuracy in each of the five organisms, while the multi-class model maintains high accuracy and is more robust in generalisation. We performed an ablation study to quantify the impact of different sequence record features, finding that features derived from meta-data, sequence identity, and alignment quality impact performance most strongly. The study demonstrates machine learning can be an effective additional tool for de-duplication of genomic sequence databases. All Data are available as described in the supplementary material.

## Introduction

Duplication is a central data quality problem, impacting the volume of data that must be processed during data curation and computational analyses and leading to inconsistencies when contradictory or missing information on a given entity appears in a duplicated record. In genomic sequence databases, duplication has been a recognised issue since the 1990s [1]. It is now of even greater concern, due to the rapid growth and wide use of sequence databases, with consequences such as redundancy, repetition in BLAST search results, and incorrect inferences that may be made from records with inconsistent sequences or annotations. It is therefore valuable to develop methods that can support detection, and eventually flagging or removal of, duplicates.

Existing duplicate detection methods in sequence databases fall into two categories. One category defines duplicates using simple heuristics. These methods are very efficient, but may be overly simplistic, resulting in high levels of both false positive and false negative detections. For example, records with default 90% sequence identity are considered as duplicates in methods such as CD-HIT [2]. Those methods can efficiently cluster sequences into groups. However, at least two questions remain: (1) Are records with high sequence identity really duplicates? This is critical when database curators merge records; only true duplicates should be merged. (2) Is a sequence identity threshold, e.g. 90%, a meaningful constant for all organisms? As we explain later, duplicates in one organism may have different types and may further differ between organisms. The other category aims to detect duplicates precisely, based on expert curated duplicate sets. However, the datasets consulted have been small and are often not representative of the full range of duplicates. For instance, the dataset in one representative method only has duplicates with exact sequences [3], whereas duplicates could be fragments or even sequences with relatively low identity, as we illustrate in this paper.

In this work, we consider an approach designed for precise detection, but tested on a large volume of representative data. Specifically, we explore the application of supervised learning to duplicate detection in nucleotide databases, building on a large collection of expert curated data that we have constructed. We make the following contributions: (1) we explore a supervised duplicate-detection model for pairs of genomic database records, proposing a feature representation based on 22 distinct attributes of record pairs, testing three learning algorithms, and experimenting with both binary and multi-class classification strategies, (2) we train and test the models with a data set of over one million expert-curated pairs across five organisms, and (3) we demonstrate that our proposed models strongly outperform a genomic sequence identity baseline. All the data we used in the study is publicly available.

## Materials and Methods

### Background

The volumes of data deposited in databases have brought tremendous opportunity for data-driven science and decision making, yet significant data quality issues have emerged. General data quality surveys have identified five main data quality problems: inconsistency (contradictory data arising from one or more sources); duplication (more than one record referring to the same entity); inaccuracy (errors); incompleteness (missing information), and obsolescence (out-of-date values) [4]. These issues can have serious impacts. Credit-card fraud is an illustrative case of duplication where different individuals may illegally use the same identity, with significant implications; the New South Wales state government in Australia reported the cost of such fraud to total over $125 million in the state from 2008 to September 2013 [5].

Data quality in bioinformatics databases is likewise an ongoing problem. In the 1990s, researchers warned that data quality concerns were emerging and should be seriously considered, in spite of efforts to annotate new genome data as quickly as possible [6]. They observed a range of data quality issues in genomic databases such as reading frame inconsistencies, missing start and stop codons, and, specifically, the presence of duplicate records [1]. Recent literature also shows that data quality issues may impact biological studies [7, 8]. Data curation is thus necessary. For example, Swiss-Prot has set up sophisticated expert curation processes to ensure high data quality as a core UniProt activity [9]. Expert curation is expensive and time-consuming, but clearly benefits the community [10]. Duplication is a direct data curation issue (typically requiring expert knowledge to identify duplicates) and also affects data curation indirectly, by increasing the amount of data that needs to be reviewed and curated.

#### Duplicate records in genomic sequence databases

Related studies have different definitions of “duplicate records”. Some consider duplicates as redundancies—records with very high or 100% similarity; for example, cd-hit and TrEMBL use 90% (by default) [2] and 100% [9], respectively. In contrast, others consider duplicates with more variations, but which are not necessarily redundancies. They may use expert curation, identifying duplicates by domain experts [3, 11]. The identified duplicates are such that both records are (close to) the same, but are not restricted to be so.

Thus the definition of “duplicate records” is context-dependent. We identify at least three relevant aspects of context:

1. Different biological databases. For example, Swiss-Prot considers duplicates as records belonging to the same gene in the same organism, whereas TrEMBL considers duplicates as records having exactly the same sequence in the same organism;
2. Different biological methods. For example, a method addressing gene-name entity recognition may consider duplicates to be records with the same literature IDs in both training and testing sets, whereas a method for detecting duplicate literature considers duplicates to be the same publications in one or more biomedical databases, including duplicate records having missing and erroneous fields and duplicate records in different or inconsistent formats;
3. Different biological tasks. For example, curation of the Pfam database labels as duplicates proteomes of the same organisms having sequence similarity over 90% and having high numbers of joint records, whereas curation of the Banana Genome Hub considers duplicates to be genes in duplicated syntenic regions [12], duplicated segments, and duplicated genes within the paralogous region.

It is, therefore, unrealistic to expect to have a single and universal definition of duplicates. Different definitions lead to different kinds of duplicates with different characteristics, and are relevant to different tasks. There is no absolute correct definition—they have different focuses or purposes. A good duplicate detection method, however, must reflect such diversity, and its performance must be tested in data sets with different duplicate types derived from multiple sources, where the test data is independent from the method [13]. In the scope of duplicate detection in biological databases, this diversity implies the need to test against various kinds of duplicates. Indeed, a simple classification of our collection of duplicates in genomic sequence databases already illustrates substantial diversity. To be robust we need to examine the performance on detection of different types and the generalisation across different organisms.

Arguably the best way to understand duplicates is via expert curation. Human review—experts checking additional resources, and applying their experience and intuition—can best decide whether a pair is a duplicate, particularly for pairs whose identity cannot be easily determined automatically [13]. The ultimate goal of an automatic system should be to model expert review to detect duplicates precisely and efficiently. Indeed, the most effective published duplicate detection methods “learn” from expert curation, using (semi-) supervised learning to build an automatic model by training from a set of expert labelled duplicates [14–16].

In this work, we take a pragmatic approach to identification of duplication. We consider duplication to have occurred when more than one nucleotide coding sequence record is cross-referenced to the same protein record through a mapping between Swiss-Prot and INSDC. This assumption satisfies the requirements of a good duplicate detection method: Swiss-Prot staff have confirmed that these nucleotide records can be considered duplicates (personal communication, Elisabeth Gasteiger) and Swiss-Prot uses sophisticated expert curation that is arguably the state-of-the-art in biocuration. The classification, as we show later, identifies different kinds of duplicates. We have collected duplicates from five organisms. Thus the method is tested against multiple duplicate types under multiple organisms.

Regardless of variation in the definitions, the impacts of duplicates are obvious. They affect the biological databases: the database may be unnessarily large, impacting storage and retrieval. They affect the biological tasks: for instance, duplicates decrease the information density in BLAST, making biased search results [17](http://www.uniprot.org/help/proteome_redundancy). They affect biocuration: wasting biocurators’ time and efforts. They affect the biological analysis: duplicates with inconsistent sequences or metadata can undermine inference and statistical analysis.

These impacts lead to the necessity for both efficient and accurate duplicate detection. Some applications need methods that are scalable in large datasets, whereas others require precise knowledge of duplicates. Both false positive (distinct pairs labelled as duplicates) and false negative (pairs that are not found) errors are problematic. For instance, merging of two records referring to the same coding sequence with inconsistent annotations may lead to incorrect prediction of protein function. We now present these two kinds of methods.

### Duplicate detection in genomic sequences databases

Approaches to identification of duplicate pairs that focus on efficiency are based on simple, heuristic criteria. Three representative methods include nrdb90, in which it is assumed that any pair with over 90% sequence identity is a duplicate, using short-word match to approximate sequence identity [18]; cd-hit, with the same assumptions as nrdb90, using substring matching to approximate sequence identity [19] (a faster version was released in 2012 [2]); and startcode, where it is assumed that “duplicates” are pairs with a thresholded edit distance (counting insertions, deletions and substitutions), using a trie data structure to estimate the possible number of edits [20].

However, recall that duplication is richer than simple redundancy. Records with similar sequences may not be duplicates and vice versa. For example, Swiss-Prot is one of the most popular protein resources in which expert curation is used. When records are merged, biocurators do not just rely on sequence identity to determine whether they are duplicates, but in many cases will manually check the literature associated with the records. In this case, priority has been given to accuracy rather than efficiency, and thus it is necessary to have accuracy-based duplicate detection methods.

Accuracy-focused duplicate detection methods typically make use of expert-labelled data to develop improved models. Such duplicate detection takes advantage of expert-curated duplicates, in one of two ways. One is to employ supervised learning techniques to train an automatic duplicate detection model [3]; the other is to employ approximate string matching such as a Markov random model [21], shortest-path edit distance [22], or longest common prefix matching [11]. However, a simple threshold for approximate string matching leads to inconsistent outcomes, as different kinds of duplicates may have different characteristics. Therefore we explore the application of machine learning to overcome these limitations, with an emphasis on coverage of duplicate diversity.

Applying (semi-) supervised learning to detection of duplicates is a promising and mature approach. Since 2000 a range of methods have been proposed [23–25]; we summarise a selection of recent duplicate detection methods using supervised learning in different domains in Table 1. These methods typically involve selection of a pair of records from a database, representing them in terms of similarity scores across selected fields of the records, and applying standard machine-learning strategies to the pairwise features, taking advantage of an expert-curated resource for training data.

**Table 1:** Representative recent supervised learning methods to detect duplicates in general domains.

Method	Domain	Expert curated set (DU + DI)	Technique(s)
[15]	Geospatial	1,927 + 1,927	DT and SVM
[26]	Product matching	1,000 + 1,000	SVM
[14]	Document Retrieval	2,500 + 2,500	SVM
[27]	Bug report	534 + 534	NB, DT and SVM
[28]	Spam check	1,750 + 2,000	SVM
[29]	Web visitor	250,000 + 250,000	LR, RF, and SVM

DU: duplicate pairs; DI: distinct pairs; NB: Naïve Bayes; DT: Decision Tree; SVM: Support Vector Machine; LR: Logistic Regression; RF: Random Forest; The dataset listed here is for supervised learning. Some work might have other datasets.

For duplicate detection in genomic sequence databases, supervised learning has received little attention, although it has been applied in other contexts such as protein function annotation [30, 31]. We have identified only one prior duplicate detection method using supervised learning [3]. That work follows essentially the approach described above, selecting 9 fields from sequence records, and computing similarity scores pairwise. The method then applies association rule mining to learn classification rules, generating the rule “Sim(Sequence) = 1.0 & Sim(Length) = 1.0 → Duplicate” as the most significant. This rule states that, if both records in a pair have the same sequence, they are duplicates.

This method has serious shortcomings. The training data set contained only labelled duplicates (no negative examples) and the method was tested on the same duplicates. In previous work, we reproduced the method based on the original author’s advice and evaluated against a sample of labelled duplicates in *Homo sapiens* [32]. The results demonstrate that the method suffers from a range of defects making it unsuitable for broader application. We did a further study applying it to an *Escherichia coli* (E. coli) dataset. The performance is still poor, due to multiple limitations. First, the training dataset only has one class (duplicates). Therefore the generated rules cannot distinguish duplicate from non-duplicate pairs. Second, some cases of field matches are absent; for example, the presence of two different values in a field is not equivalent to the case where one record has a value and the other is missing a value for that field. Third, most feature similarities are quantities in the original study, but they are all converted to labels in order to apply association rule mining. Decision trees or SVMs may be better choices in this case. Last, the labelled dataset is small and contains a narrow set of duplicate types. The dataset used in the method only has 695 duplicate pairs, where most contain exactly the same sequence. This may have led to over-fitting.

### Methods

**Fig 1:**
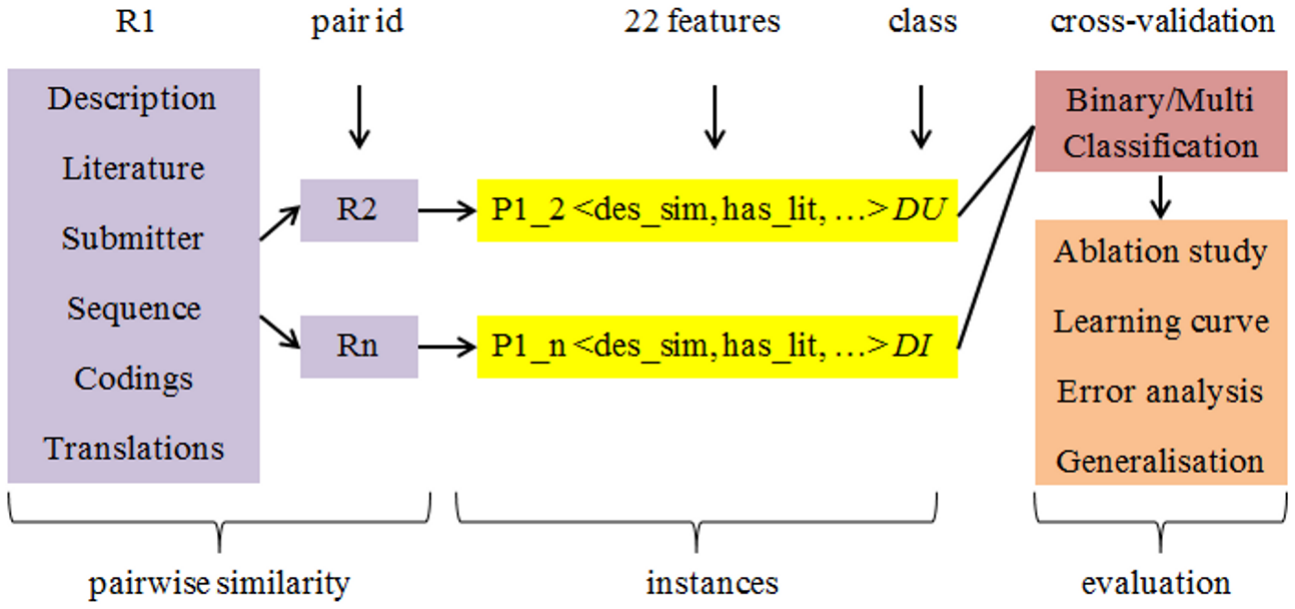
The general architecture of our approach. R: record; Pair R1 R2 and Pair R1 RN are expert labelled duplicate and distinct pairs respectively; Binary: whether a pair is duplicate or distinct; Multi: multiple duplicate types and distinct pairs; Ablation: quantify the impacts of different features; Error: quantify erroneous cases to characterise challenging cases; Generalisation: whether model can be applied to a different dataset.

Fig 1 summarises the general architecture of our approach. For each organism set in the collection, the feature similarity of labelled duplicate and distinct pairs is computed. Then a binary or multi-class model is built using Naïve Bayes, decision trees, or SVMs, and evaluated via 10-fold cross-validation. The binary model recognises two classes, duplicate or distinct, whereas the multi-class model breaks duplicates into different (sub-) types. Each organism set is designed to have balanced duplicate and distinct pairs, as for other supervised learning methods in Table 1. Note that handling of an imbalanced dataset is a distinct area in machine learning that often leads to separate work [33].

#### Data collection

For sequence databases, UniProtKB is well-known for its high-quality data. Its Swiss-Prot section is subject to detailed expert curation including a range of quality checks [30]. We used Swiss-Prot to construct a labelled dataset of nucleotide sequence record duplicates, based on the observation that duplication occurs when a protein record in UniProt cross-references more than one coding sequence record in the INSDC nucleotide databases (International Nucleotide Sequence Database Collaboration: GenBank, EMBL ENA and DDBJ: http://www.insdc.org/) [34]. We used the mapping service between Swiss-Prot and INSDC, which provides protein records and cross-referenced nucleotide coding sequence records, and collected duplicate nucleotide records for five commonly studied organisms: *Caenorhabditis elegans*, *Danio rerio*, *Drosophila melanogaster*, *Escherichia coli*, and *Zea mays*. The collections are summarised in Table 2. Finally, we randomly selected a similar number of distinct pairs for each of these organisms. To the best of our knowledge, it is the largest collection of duplicates in this domain, and larger than many non-biological duplicate reference sets. Building on the sophisticated expert curation in Swiss-Prot, the collection is also representative and reliable.

**Table 2:** Size of data collections used in our work.

Organism	Classes		Total
	DU	DI	
Caenorhabditis elegans	4,472	4,474	8,946
Danio rerio	4,942	4,942	9,884
Drosophila melanogaster	553,256	569,755	1,123,011
Escherichia coli	1,042	1,040	2,082
Zea mays	16,105	15,989	32,094

DU: duplicate pairs; DI: distinct pairs.

#### Record examples

Observing the collection, we found pairs with similar sequences that are not duplicates, and vice versa, clearly showing that simple assumptions based on sequence similarity alone are not sufficient. For example:

- Records accession AL117201 and Z81552, marked as *duplicate*, from *Caenorhabditis elegans*, and submitted by the same institute, has local identity of only 69%. The measurement procedure is summarised in the Feature computation section, according to advice from Wayne Mattern of the NCBI BLAST team (personal communication). These are different clones for the same protein record Q9TW67;
- Records accession U51388 and AF071236, marked as *duplicate*, from *Danio rerio*, and submitted by different groups, have local identity only 71%. These are different fragments for the same protein record P79729;
- Records accession X75562 and A07921, marked as *distinct*, from *Escherichia coli*, and one submitter not specified (not provided in GenBank required format shown in Feature computation), have local identity of 100%, and length ratio of 72%. These are similar coding sequences but for different proteins;
- Records accession FJ935763 and M58656, marked as *distinct*, from *Zea Mays*, and one submitter not specified, have local identity 100%, length ratio 98%. These are similar coding sequences but for different proteins.

#### Feature selection and representation

We selected features that may distinguish duplicates from distinct pairs. A genomic sequence database record consists of two components: meta-data, such as record description; and sequence. We extracted 22 features as shown in Table 3 from the nucleotide records. These features play different roles and cover distinct cases. We describe them based on the GenBank format documentation (http://www.ncbi.nlm.nih.gov/Sitemap/samplerecord.html) and explain why we selected them below.

**Table 3:** All features used in our method.

Feature	Definition	Type	Range	Example
Description	Description similarity ratio	N	[0,1]	0.35
Has_Literature	Record has literature	C	(Yes, No)	Yes
Literature	Literature similarity ratio	N	[0,1]	0.50
Submitter	Same submitters	C	(S, D, NA),	Same
Length	Length ratio	N	[0,1]	0.23
Has_HITS	Has HITS	C	(Yes, No)	Yes
Identity	Sequence local identity	N	[0,1]	0.90
AP	Aligned proportion	N	[0,1]	0.68
Expect_Value	Expect value	N	≥ 0	0.0001
Over_Threshold	Expect value over threshold	C	(Yes, No)	No
Has_CDS	Has CDS	C	(Yes, No)	Yes
CDS_HITS	Has HITS between CDS	C	(Yes, No)	No
CDS_Identity	CDS local identity	N	[0,1]	0.95
CDS_AP	CDS alignment proportion	N	[0,1]	0.80
CDS_Expect	Expect value of CDS	N	≤ 0	1.2
CDS_Threshold	CDS expect value over threshold	C	(Yes, No)	Yes
HAS_TRS	Has TRS	C	(Yes, No)	No
TRS_HITS	Has HITS between TRS	C	(Yes, No)	No
TRS_Identity	TRS local identity	N	[0.1]	0.71
TRS_AP	TRS alignment proportion	N	[0,1]	0.32
TRS_Expect	Expect value of TRS	N	≤ 0	0.3
TRS_Threshold	TRS expect value over threshold	C	(Yes, No)	No

N: numerical (quantitative) variable; C: categorical (qualitative) variable; HITS: BLAST HITS; AP: alignment proportion; CDS: coding sequence extracted from the whole sequence; TRS: translations of CDS.

*Description* is specified in the record *DEFINITION* field, where submitters manually entered a few words to describe the sequence in the record. Similar records may be described using similar terminologies. Use of approximate matching finds records with shared vocabulary.

*Has_Literature*, *Literature*, and *Submitter* are specified in the record *REFERENCE* field. The first two refer to publications where record authors introduced the sequence represented by the record. *Has_Literature* indicates whether or not a record has at least one literature reference. This can distinguish pairs that do not have literature references from pairs whose literature similarity is 0. *Submitter* describes the details of the submitter. It has a special label “Direct Submission”. We have observed that duplicates may be submitted by different groups or by the same groups, or submitter details may not be provided. These features can potentially find similar records discussed in related literature.

*Length*, *Has_HITS*, *AP*, *Identity*, *Expect_Value*, and *Over_Threshold* are derived from the record *ORIGIN* field, the complete sequence of the record. *Length* is the sequence length ratio of a pair of sequences. The rest is based on BLAST output. *Identity* defines local sequence identity of the pair. The rest reflects the quality of the alignment: *AP* (aligned proportion) estimates global coverage of the pair without doing actual global alignment; *Expect_Value* measures whether the alignment is “significant” and *Over_Threshold* is whether the expected value is over the defined threshold. We discuss these further in Feature computation.

All the features starting with “CDS” are from the record *CDS* field, whereas the features starting with “TRS” are from the record *translation* field. GenBank specifies coding sequence regions in the *CDS* field. For each *CDS*, its translation is specified in *translation*, a subfield of *CDS*. The remainder of the features related to “CDS” or “TRS” are similar to the above features, but for the whole record sequence. For example, *CDS_AP* is the alignment proportion for coding region, whereas *AP* is for the whole sequence. Note that a record might have multiple “CDS” and “TRS” subfields, so “CDS” may be just a subsequence. “CDS” and “TRS” related features may be useful for finding difficult cases in which a distinct pair has high overall sequence identity, but relatively different coding regions and translations.

#### Feature computation

Feature similarities are calculated pairwise using different methods. Any feature starting with “HAS” is used to check whether the corresponding field exists. It is denoted as “No” if a record in a pair does not have that field. We explain the rest of the features as follows.

**Description similarity:** We applied elementary natural language processing for the *Description* field. This included tokenising, splitting the text into words, and lowering the case; removing stop words; lemmatising, or reducing a word to its base form, such as “encoding” to “encode”; and representing the tokens as a set. For the *Description* similarity of a pair, we calculated the Jaccard similarity of their corresponding token sets. This measure calculates the number of shared elements over two sets dividing by the total number of elements. This would find descriptions with similar tokens in different orders.

**Literature similarity:** For *Literature* similarity, we used a rule-based comparison: (1) If both literature fields contain *PUBMED* IDs (the identifier of linked PubMed), then direct Boolean matching is applied; (2) If both literature fields have a *JOURNAL* field, then the titles will be compared using the text processing method above. If neither of these two cases apply, the author names will be compared using Jaccard similarity.

**Submitter similarity:** We measured *Submitter* strictly following INSDC policy. Records can be modified or updated if one original submitter agrees (http://www.ebi.ac.uk/ena/submit/sequence-submission#how_to_update). We used three labels: “SAME” for pairs having at least one common submitter; “DIFFERENT” for not having any common submitters; and “N/A” when at least one record does not have submitter information.

**Sequence, coding regions, and translation similarity:** Sequence, coding region, and translation-related features are all computed using a similar approach. We used NCBI BLAST (version 2.2.30) [35] and parameter settings recommended by NCBI staff (personal communication, Wayne Mattern) to produce reliable outcomes. We used the *bl2seq* application for pairwise sequence alignment. We disabled the dusting parameter and selected the smallest word size (which was 4), to achieve high accuracy in the output. Features can then be derived from the alignment output: *Identity* is local sequence identity; *Expect_Value* is the E-value in the output; *Has_HITS*: whether it has “HITS” in the output (BLAST uses “NO HITS” when no significant similarity found in a pair). *Over_Threshold* identifies whether the E-value in the output is greater than 0.001. *AP* (alignment proportion) was calculated using Formula 1. This estimates global sequence identity rather than performing exact global alignment.

$$AP=\frac{len(I)}{max(len(D),len(R))}$$ (1)

where *D* and *R* are sequences of a pair being compared; *I* is a sequence comprised of locally aligned identical bases; and *len*(*S*) is the length of a sequence *S*.

For coding region and translation-related features, essentially the same method is used. The minor differences are: the task is *blastp*, the minimum word size is 2, and no dusting parameter is used for translations (proteins). Since one record may have multiple coding regions, we selected only the first one and its translations in this work.

#### Classification

We explore two approaches to the genomic record pair classification task, as well as considering the cross-species generalisation of the models. We evaluate these methods using 10-fold cross-validation, and compare with a simple baseline method, *Seq90*, in which a pair is considered to be a duplicate if their *Identity* and *Length* similarity is no less than 90%. We note that a majority class baseline (ZeroR) is not relevant here; due to the balanced distribution of the labels in the data, its performance would be 0.5.

**Binary classification, duplicate vs. distinct:** This model aims to classify into two classes: *duplicate* and *distinct* pairs. We employed Naïve Bayes, decision trees, and SVM to build models. For the first two we used default implementations in WEKA [36] and LIBSVM [37] for SVM. We followed the LIBSVM authors’ guidelines; for instance, we scaled the data for accuracy [38]. We built models for each organism set and used 10-fold cross-validation to assess the stability of the models.

**Multi-class classification:** Duplicates have different kinds with distinct characteristics. Considering all kinds as a monolithic class may drop the performance due to differences in features that are relevant to different kinds. We thus built multi-class models that treat each kind of duplicate as a separate class (in addition to the “distinct” class). Naïve Bayes and decision trees inherently perform multi-class classification. LIBSVM uses a one-to-one (comparing each class pairwise) approach by default for classifying into multiple classes [37].

We subclassified duplicates based on identity and alignment coverage:

- ***ES*** (exact sequence): approximate or exact sequences, pairs with both *Identity* and *AP* not less than 0.9;
- ***NS*** (non-significant alignments): pairs with either *Expect_value* is over 0.001 or *Has_HITS* is “No”. *Expect_value* itself does not measure the sequence identity, but it is arguably the most important metric for assessing the statistical significance of the alignment (with the exception of short sequences). Duplicate pairs in this class could be pairs with relatively different sequences, or with similar sequences but not similar enough to be the part of *ES* class;
- ***EF*** (exact fragment): approximate or exact fragments, pairs satisfying the threshold and having “HITS”, but below the criteria of *ES*.

Table 4 presents these categories with their frequency in each organism data set. It shows that different organisms have differing distributions of duplicate types. For instance, *EF* has the highest prevalence in *Zea Mays*, whereas in *Drosophila melanogaster* *ES* is the most prevalent. This demonstrates the complexity of duplication. Supervised learning within an organism is sensitive to the patterns within that organism.

**Table 4:** Different classes of duplicates used in multi-class.

Organism	Duplicate types		
	EF	ES	NS
Caenorhabditis elegans	3,074	1,243	155
Danio rerio	4,017	836	89
Drosophila melanogaster	115,643	307,305	130,308
Escherichia coli	855	170	17
Zea mays	10,942	5,104	59

EF: close to or exact fragments; ES: close to or exact sequences; NS: non-significant alignments.

## Results and Discussion

**Binary classification** The binary classifiers have high performance, as shown in Table 5. Most have over 90% accuracy and all substantially outperform the Seq90 sequence similarity baseline. The poor performance of this baseline clearly demonstrates that a single simple assumption is inadequate to model duplication. While in *Drosophila melanogaster* and *Zea Mays*, where duplicates often have similar or same sequences, Seq90 achieves over 65% accuracy (though some precision and recall values are still low), it cannot handle other organisms where duplication is more complex. In fact, for easy cases, most methods easily achieve high performance; note for example the near-100% accuracy of decision trees in these two organisms. Similarity, the AUROC of the three machine learning classifiers is above 0.89, while the AUROC for Seq90 does not exceed 0.75, showing that they have reliable performance with less bias than the simple sequence baseline.

**Table 5:** Performance for binary classifiers under each organism (AUROC = area under the receiver operator characteristic curve).

Organism	Precision		Recall		AUROC		Accuracy
	DU	DI	DU	DI	DU	DI	
Caenorhabditis							
*Seq90*	0.955	0.586	0.302	0.986	0.644	0.644	0.644
*Naïve Bayes*	0.974	0.730	0.636	0.983	0.910	0.910	0.809
*Decision tree*	**0.986**	**0.975**	**0.975**	**0.986**	**0.987**	**0.987**	**0.981**
*SVM*	0.926	0.921	0.920	0.926	0.923	0.923	0.923
Danio							
*Seq90*	0.814	0.547	0.210	0.952	0.566	0.544	0.581
*Naïve Bayes*	**0.985**	0.694	0.562	**0.992**	0.929	0.929	0.777
*Decision tree*	0.964	0.952	0.951	0.965	**0.984**	**0.984**	**0.958**
*SVM*	0.834	**0.971**	**0.976**	0.806	0.891	0.891	0.891
Drosophila							
*Seq90*	0.947	0.702	0.576	0.969	0.754	0.694	0.775
*Naïve Bayes*	0.991	0.976	0.975	0.992	0.984	0.986	0.983
*Decision tree*	**0.999**	**0.999**	**0.999**	**0.999**	**0.999**	**0.999**	**0.999**
*SVM*	0.993	0.995	0.995	0.993	0.994	0.994	0.994
Escherichia							
*Seq90*	0.892	0.550	0.205	0.975	0.581	0.549	0.589
*Naïve Bayes*	**0.990**	0.864	0.845	0.991	**0.987**	**0.989**	0.918
*Decision tree*	0.979	**0.983**	0.983	**0.979**	0.980	0.980	**0.981**
*SVM*	0.960	**0.983**	**0.984**	0.959	0.971	0.971	0.971
Zea							
*Seq90*	0.921	0.608	0.381	0.967	0.662	0.604	0.673
*Naïve Bayes*	0.996	0.976	0.976	0.996	0.987	0.989	0.986
*Decision tree*	**0.999**	**0.998**	**0.998**	**0.998**	**0.998**	**0.998**	**0.998**
*SVM*	0.996	0.993	0.993	0.996	0.995	0.995	0.995

DU: duplicate pairs; DI: distinct pairs; Accuracy is for all the instances.

**Learning curve** The performance is reasonably good in all of these organisms. An interesting question is, given a classifier, how much training data is sufficient to achieve peak performance? Too little training data will not be sufficient; too much training data wastes time. As an additional evaluation, we measured the learning curve of classifiers. For 10-fold cross validation, each time we randomly sampled *X*% of the 9-fold training data, trained the classifier with the sampled data, and tested against the same fold of testing data. We increased *X* exponentially to demonstrate the growth trend across orders of magnitude. (Specifically, starting from 1%, we increased each time by multiplying by $\sqrt[{5}]{10}$, up 100% was reached.) For each sample we recorded five metrics: overall accuracy, and the precision and the recall for both *DU* and *DI*. Each measurement was repeated 20 times with different random seeds.

**Fig 2:**
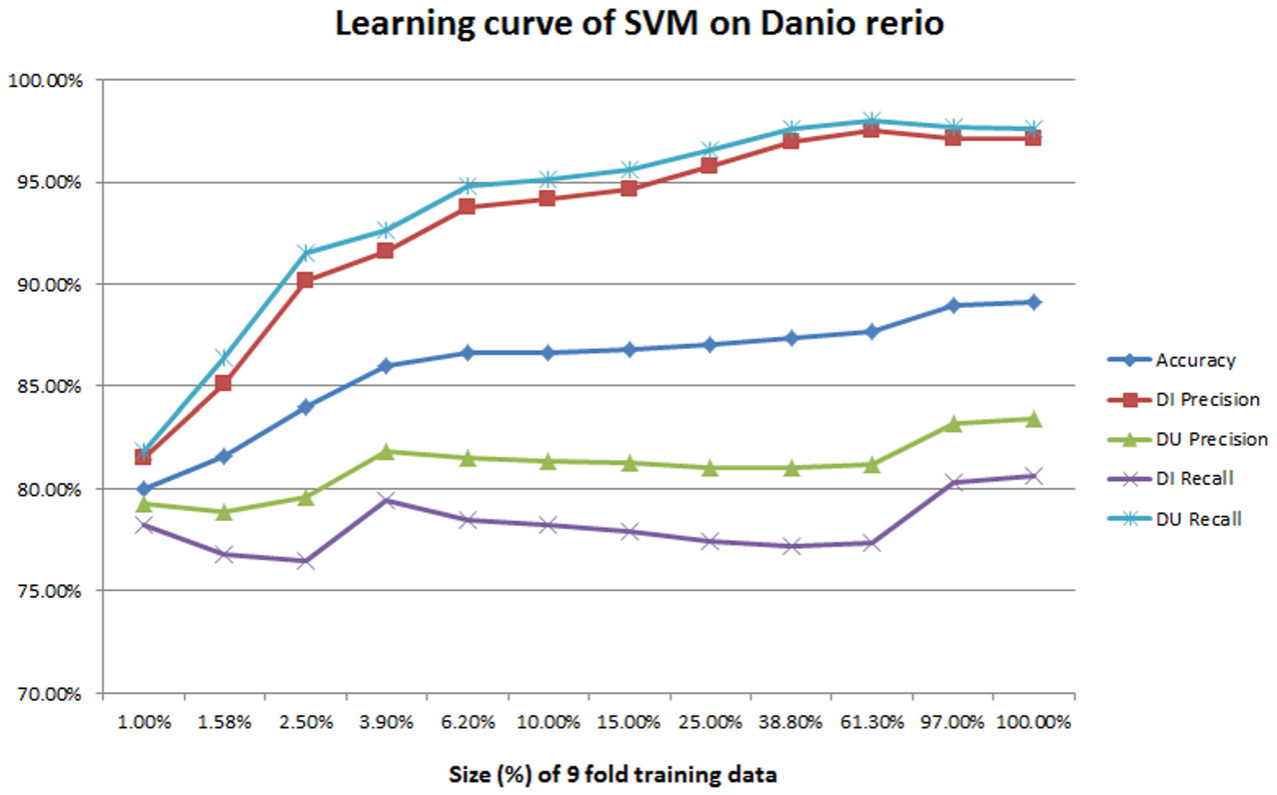
The learning curve of SVM on *Danio rerio*.

**Fig 3:**
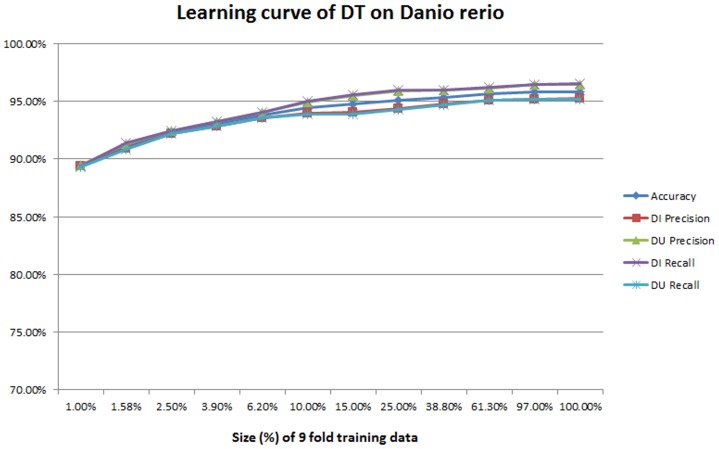
The learning curve of decision trees on *Danio rerio*.

Figs 2 and 3 illustrate the learning curve of SVMs and decision trees on Danio rerio. The same measurements on Escherichia coli are provided in S1 and S2 Figs. We made two observations: First, for SVMs, when the training size is small, the performance is low. For example, the recall of *DU* is less than 70% when the sample is 1% of the training space. The performance improves considerably as the training dataset size increases. It reaches the peak before using 100% of the training data, but the volume of training data required depends on the organisms: for example 61.30% (6058 records) for *Danio rerio* but only 6.20% (129 records) for Escherichia coli. This means that SVMs may not need such large sets of data to achieve the best performance. Second, for decision trees, when the training dataset size is small, the performance is already reasonably good—close to 90% for all the five metrics. This means we extracted all the important features and worked out the dominant features so that the tree is well-split even when the training dataset size is small. We did an ablation study later to quantify which features are important as a further investigation. However, performance continues to improve as training set size is increased, and overall, compared to SVMs, more data seems to be required for peak performance.

**Ablation study** We quantified the impacts of different kinds of features via an ablation study. We measured the performance of five feature sets; results are summarised in Table 6.

- *Meta*: meta-data features including *Description* and *Literature* related features;
- *Seq*: sequence features: *Length* and *Identity*;
- *SQ*: features in *Seq* plus features checking alignment quality such as *Expect_value*;
- *SQC*: features in *SQ*, plus *CDS* and *TRS* related features; and
- *SQM*: a combination of *SQ* and *Meta*.

**Table 6:** Ablation study of record features for duplicate classification.

Organism	Meta		Seq		SQ		SQC		SQM		All	
	Pre	Rec	Pre	Rec	Pre	Rec	Pre	Rec	Pre	Rec	Pre	Rec
Caenorhabditis												
*Naïve Bayes*	0.633	0.628	0.714	0.714	0.872	0.833	0.849	0.808	**0.899**	**0.880**	0.852	0.809
*Decision tree*	0.815	0.730	0.816	0.814	0.971	0.971	0.979	0.979	0.980	0.980	**0.981**	**0.981**
Danio												
*Naïve Bayes*	0.656	0.622	0.696	0.657	0.817	0.766	**0.839**	0.775	0.831	**0.797**	**0.839**	0.777
*Decision tree*	0.815	0.730	0.816	0.814	0.971	0.971	0.979	0.979	**0.980**	**0.980**	0.958	0.958
Drosophila												
*Naïve Bayes*	0.945	0.941	0.719	0.718	0.860	0.827	0.882	0.849	0.973	0.973	**0.983**	**0.983**
*Decision tree*	0.951	0.950	0.950	0.950	0.996	0.996	0.998	0.998	**0.999**	**0.999**	**0.999**	**0.999**
Escherichia												
*Naïve Bayes*	0.778	0.654	0.842	0.820	**0.979**	**0.979**	0.937	0.930	0.972	0.972	0.927	0.918
*Decision tree*	0.719	0.717	0.842	0.836	**0.982**	**0.982**	0.981	0.981	0.981	0.981	0.981	0.981
Zea												
*Naïve Bayes*	0.894	0.881	0.882	0.855	0.987	0.986	0.987	0.986	0.984	0.984	0.986	0.986
*Decision tree*	0.961	0.960	0.965	0.965	0.997	0.997	**0.998**	**0.998**	**0.998**	**0.998**	**0.998**	**0.998**

Pre: average precision for two classes (DU and DI); Rec: average recall; Meta: meta-data features; Seq: sequence identity and length ratio; Q: alignment quality related features, such as Expect_value; SQ: combination for Seq with Q; C: coding regions related features, such as CDS_identity; SQC: combination for Seq, Q and C; SQM: Seq, Q and Meta All: all eatures.

We find that meta-data features alone are competitive with the simple sequence baseline shown in Table 5. The *Meta* feature set has over 60% precision and recall in all organisms, and over 88% in “easy” organisms *Drosophila melanogaster* and *Zea Mays*. Considering that meta-data are just short record fields, the computational cost of using these features is lower than that of full sequence alignment. Therefore, meta-data may be able to be used as a filter to eliminate clearly distinct pairs. In duplicate detection, this approach is called *blocking* [39]. Given that these features have reasonable performance, we will apply meta-data blocking in future work.

The sequence field is arguably the most critical field, but we see benefit from including the actual similarity value. Existing studies focused either on a simple fixed identity threshold, or only use sequence identity together with a length ratio. Considering the quality of sequence alignment increases the performance of these classifiers by about 15% compared to considering sequence identity only (*Seq+Qua* cf. *Seq*). It means that features from Qua validate alignment quality, ensuring reliable sequence coverage and meaningfulness of sequence identity. Using them enables identification of difficult cases such as distinct pairs with high identity but low reliability.

Coding region related features may lower the performance. *SQC* has lower performance in most cases than *SQ*. This may be because we only compared the first coding regions of a pair and their translations. Performance may improve when considering all the coding regions and translations, but with a trade-off for longer running time due to the computational requirements of calculating those features.

The best feature set is *SQM*. It has competitive performance with all features and is higher in many cases. This again shows that meta-data has a vital role: not only can it be used in blocking for efficiency, it also facilitates accuracy. Notice here that records are from INSDC; UniProt makes more abundant meta-data annotations on records. Thus we believe meta-data will be even more useful when detecting protein record duplicates.

**Validating the method in *Mus Musculus* dataset** As we are gradually collecting duplicate records in different organisms, so far the collection does not contain mammal datasets. However they are important for biological and biomedical studies. Therefore we applied the exact method in *Mus Musculus* dataset as an example. The collection consists of 244,535 duplicate pairs and 249,031 distinct pairs, using the same collecting data procedure. We used the best feature set *SQM* and compared the performance of the techniques. The results are consistent with what we have found in the existing collection. Using simple sequence identity can only achieve 64%. Our methods outperform the baseline significantly: all of the adopted machine learning techniques have the accuracy over 90%, particularly decision trees have over 97%. The results clearly show the method is generalised well and has potential to be applied in mammal datasets.

The detailed results are summarised in S1 Table. We also provide all the IDs of the *Mus Musculus* dataset.

**Error analysis** We also analysed erroneous classified instances. Table 7 summarises mistakes made by Naïve Bayes in five organisms. The corresponding analysis for decision trees is in S2 Table. For both false positives (distinct pairs classified as duplicates) and false negatives (duplicates classified as distinct), we measured average similarity for all numerical features. Some challenging cases are revealed.

**Table 7:** Error analysis: average feature similarity for error cases on Naïve Bayes.

	Caenorhabditis		Danio rerio		Drosophila		Escherichia coli		Zea mays	
Feature	FP	FN	FP	FN	FP	FN	FP	FN	FP	FN
#Instances	1644	72	2167	39	13879	4844	161	9	390	66
Description	0.322	0.320	0.293	0.372	0.250	0.515	0.147	0.172	0.216	0.428
Literature	0.115	0.027	0.440	0.243	0.031	0.471	0.003	0.000	0.013	0.232
Length	0.191	0.567	0.165	0.659	0.143	0.704	0.151	0.556	0.207	0.720
Identity	0.936	0.902	0.954	0.902	0.974	0.854	0.983	0.924	0.962	0.866
AP	0.015	0.018	0.008	0.032	0.027	0.060	0.037	0.167	0.054	0.277
Expect_Value	0.012	0.109	0.019	0.031	0.168	0.365	0.037	0.020	0.055	0.001
CDS_Identity	0.881	0.882	0.924	0.888	0.893	0.852	0.906	0.921	0.868	0.840
CDS_AP	0.018	0.022	0.006	0.032	0.020	0.072	0.022	0.146	0.009	0.413
CDS_Expect	0.458	0.348	0.596	0.299	1.126	0.36	0.753	0.589	0.614	0.056
TRS_Identity	0.403	0.512	0.392	0.345	0.426	0.424	0.430	0.548	0.540	0.840
TRS_AP	0.020	0.042	0.020	0.408	0.032	0.130	0.030	0.262	0.027	0.463
TRS_Expect	2.456	1.312	1.630	0.408	2.061	1.404	1.799	0.144	3.227	0.257

#Instances: number of instances; FP: false positives, distinct pairs classified as duplicates; FN: false negatives, duplicates classified as distinct pairs; Feature names are explained in Table 3; Numbers are averages, excluding pairs not have specific features.

For false positives, challenging cases include distinct pairs with relatively high meta-data similarity, high sequence identity but high expected value—for pairwise BLAST, high values in general indicate that the reported identity is not promising, so, under these cases, even though the reported identity is high, we cannot trust it. We found that false positives (distinct pairs) in three organisms have similar or higher meta-data and sequence similarity than false negatives (duplicate pairs). Even with quality-related features, these cases will be extremely difficult for any classifier.

Challenging false negatives include duplicate pairs with low meta-data and sequence similarity, with relatively low expected values. Low expect values indicate that the reported identity is promising, so, in these cases, duplicate pairs indeed have relatively low sequence identity, making them difficult to detect. False negatives in two organisms only have around 85% local identity with quite different lengths, meaning that the global identity will be much lower. We believe that these are most difficult duplicate instances to find.

State-of-art duplicate detection methods employ expert review for difficult cases [40]; this approach clearly has potential application in sequence database duplication as well. In general, the supervised methods are able to reliably categorise at least 90% of pairs, and our analysis has helped to identify specific feature combinations of pairs that could be pushed to a human for final resolution. Such an approach could greatly streamline data quality curation processes and achieve substantial higher reliability than simple heuristics.

Table 8 shows the performance of multi-class classifiers. In general multi-class classification is more complex than binary and thus it is hard to achieve the same or better performance. Despite this, the results show that the multi-class models maintain almost the same performance as binary classification, and even better in some organisms.

**Table 8:** Performance for multi-class classifiers under each organism.

Organism	Precision				Recall				AUROC				Accuracy
	EF	ES	NS	DI	EF	ES	NS	DI	EF	ES	NS	DI	
Caenorhabditis													
*Naïve Bayes*	0.968	0.956	0.217	0.750	0.559	0.997	**0.671**	0.904	0.984	0.999	0.882	0.930	0.795
*Decision tree*	**0.981**	**1.000**	**0.980**	**0.974**	**0.980**	**1.000**	0.626	**0.986**	**0.996**	**1.000**	**0.934**	**0.989**	**0.980**
*SVM*	0.900	0.938	0.946	0.938	0.905	0.999	0.568	0.930	0.926	0.994	0.784	0.934	0.925
Danio													
*Naïve Bayes*	**0.974**	0.803	0.431	0.705	0.458	0.990	0.281	**0.985**	0.943	0.999	**0.932**	0.930	0.765
*Decision tree*	0.954	**1.000**	**0.700**	0.955	**0.958**	**1.000**	**0.315**	0.961	**0.989**	**1.000**	0.888	**0.983**	**0.957**
*SVM*	0.803	0.860	0.000	**0.968**	0.955	0.999	0.000	0.810	0.897	0.992	0.500	0.892	0.878
Drosophila													
*Naïve Bayes*	0.939	**1.000**	0.973	0.978	0.909	0.987	0.983	0.989	0.992	**1.000**	0.995	0.995	0.980
*Decision tree*	**0.998**	**1.000**	**0.999**	**0.999**	**0.998**	**1.000**	**0.996**	**0.999**	**1.000**	**1.000**	**0.999**	**0.999**	**0.999**
*SVM*	0.991	0.998	0.978	0.995	0.984	0.999	0.986	0.994	0.992	0.999	0.992	0.992	0.993
Escherichia													
*Naïve Bayes*	**0.980**	0.994	**0.129**	0.922	0.911	0.971	**0.235**	0.966	**0.992**	0.995	**0.811**	**0.982**	0.938
*Decision tree*	0.977	**1.000**	0.000	**0.982**	**0.998**	**1.000**	0.000	**0.979**	0.989	**1.000**	0.762	0.978	**0.980**
*SVM*	0.909	0.962	0.000	0.983	0.994	0.753	0.000	0.959	0.962	0.875	0.500	0.971	0.949
Zea													
*Naïve Bayes*	0.983	0.758	0.038	0.984	0.824	0.979	**0.695**	0.939	0.984	0.997	0.962	0.991	0.906
*Decision tree*	**0.999**	**0.999**	0.881	**0.998**	**0.999**	**1.000**	0.627	**0.998**	**0.999**	**1.000**	0.875	**0.998**	**0.998**
*SVM*	0.979	0.948	**1.000**	0.994	0.972	0.967	0.017	0.996	0.980	0.978	0.508	0.995	0.981

EF: close to or exact fragments; ES: close to or exact sequences; NS: non-significant alignments; Accuracy is for all the instances.

**Binary v.s. Multi-class** To compare the performance of binary and multi-class model in terms of detecting different duplicate types, we calculated the relative accuracy for each duplicate type. As a binary classifier only classifies whether a pair is duplicate or distinct, we considered that it correctly identifies a duplicate type as long as it correctly classifies it as a duplicate. For example, if a pair is *EF* and it is classified as a duplicate, it will be considered as correct. For fair evaluation of the multi-class classifier, so long as it classifies a duplicate pair as one of the duplicate types, we consider it as correct. For example, if it classifies a *ES* pair as *EF*, it is considered correct since it has identified a duplicate.

**Fig 4:**
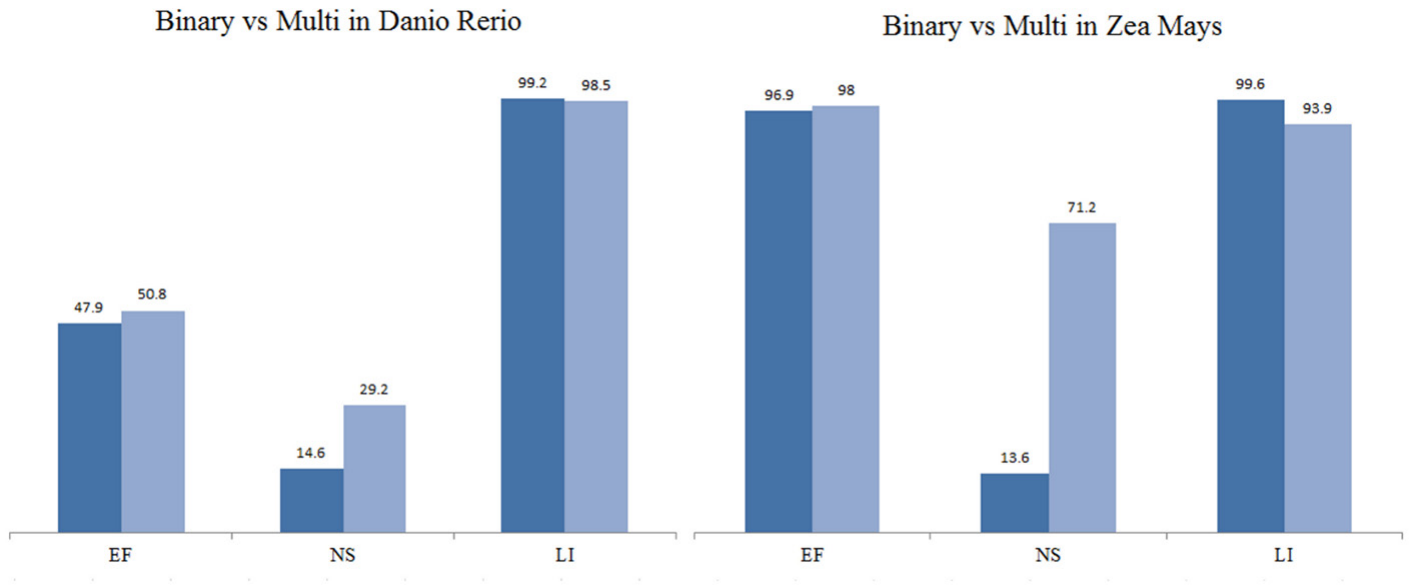
Performace of binary and multi class Naive Bayes in 2 organisms; EF: close to exact fragments; NS: non-significant alignments; DI: distinct pairs; Y axis is accuracy(%).

Fig 4 compares the performance of binary and multi-class Naïve Bayes Classifiers in *Danio Rerio* and *Zea Mays* as examples, and the confusion matrix for *Zea Mays* is also provided in Table 9 for the binary classifier and in Table 10 for the multi-class classifier. Additional results are in S3 Table. We found that multi-class Naïve Bayes improves the performance of detecting *EF* a little, boosts the performance for *NS*, and lowers the performance for *DI*. The confusion matrix shows that the binary model detected 390 duplicate pairs incorrectly, 339 of which are *EF* and 51 are *NS*. In contrast, the multi-class model only classified 223 *EF* and 17 *NS* incorrectly. While it classified some of *EF* to *ES* and *NS*, they are still duplicate categories rather than *DI*. Notice that *Zea Mays* has 59 *NS* cases in total; the binary model only got 8 correct, whereas the multi-class gets 41 cases correct. Therefore the multi-class model has potential to detect difficult duplication cases more precisely. We also observed a trade-off: it classified distinct pairs less accurately than the binary model. It confused some distinct pairs with *NS* as both types have relatively low sequence identity.

**Table 9:** Confusion matrices for Naïve Bayes in Zea Mays; binary classifier.

	DU	DI
DU	15,715	390
DI	66	15,923

339 EF and 51 NS

**Table 10:** Confusion matrices for Naïve Bayes in Zea Mays; multi-class.

	EF	ES	NS	DI
EF	9,013	1,595	111	223
ES	105	4,999	0	0
NS	1	0	41	17
DI	53	1	9,16	15,019

**Fig 5:**
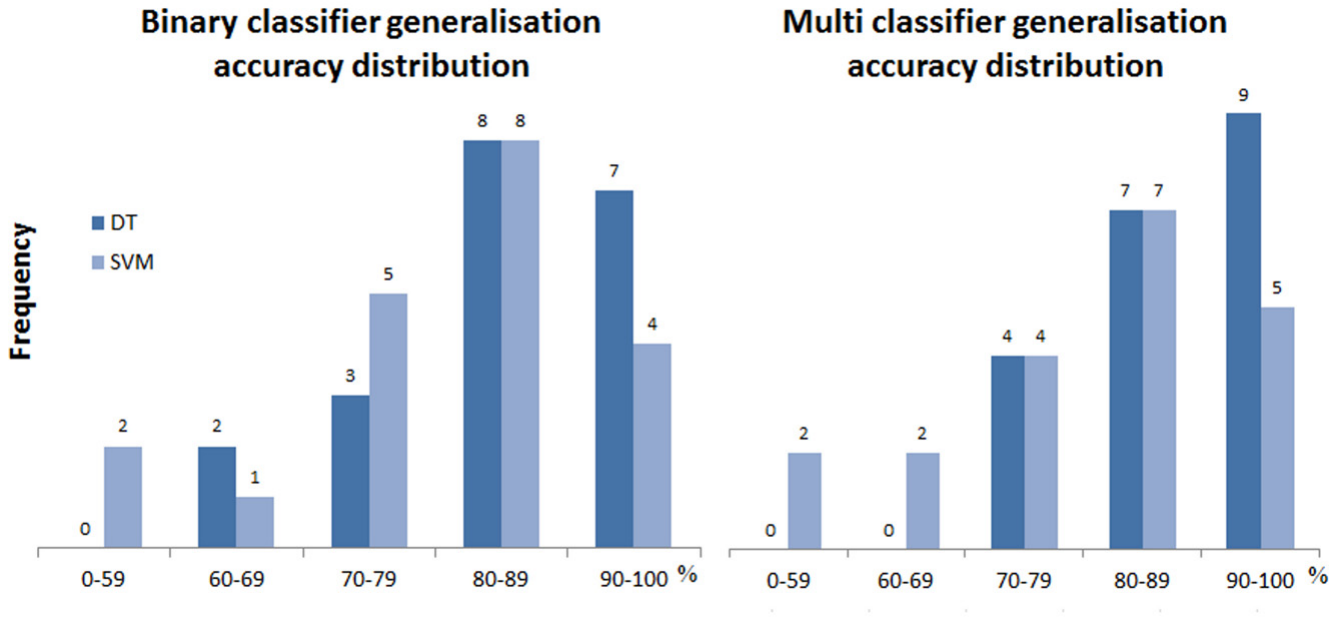
Distribution of accuracy for binary and multi-class classifier in generalisation evaluation. The left chart is for binary and the right for multi-class classification. The X axis in both refers to accuracy (%) range. The Y axis stands for frequency in specific accuracy range.

**Generalisation** We evaluated the generalisation of binary and multi-class models across organisms. For a classifier trained from one organism, we applied it to each of the remaining organisms, so that there are twenty pairs of results in total. Details are in S4 and S5 Tables. Fig 5 outlines the accuracy distribution for both the binary and multi-class decision tree and SVM models.

**Fig 6:**
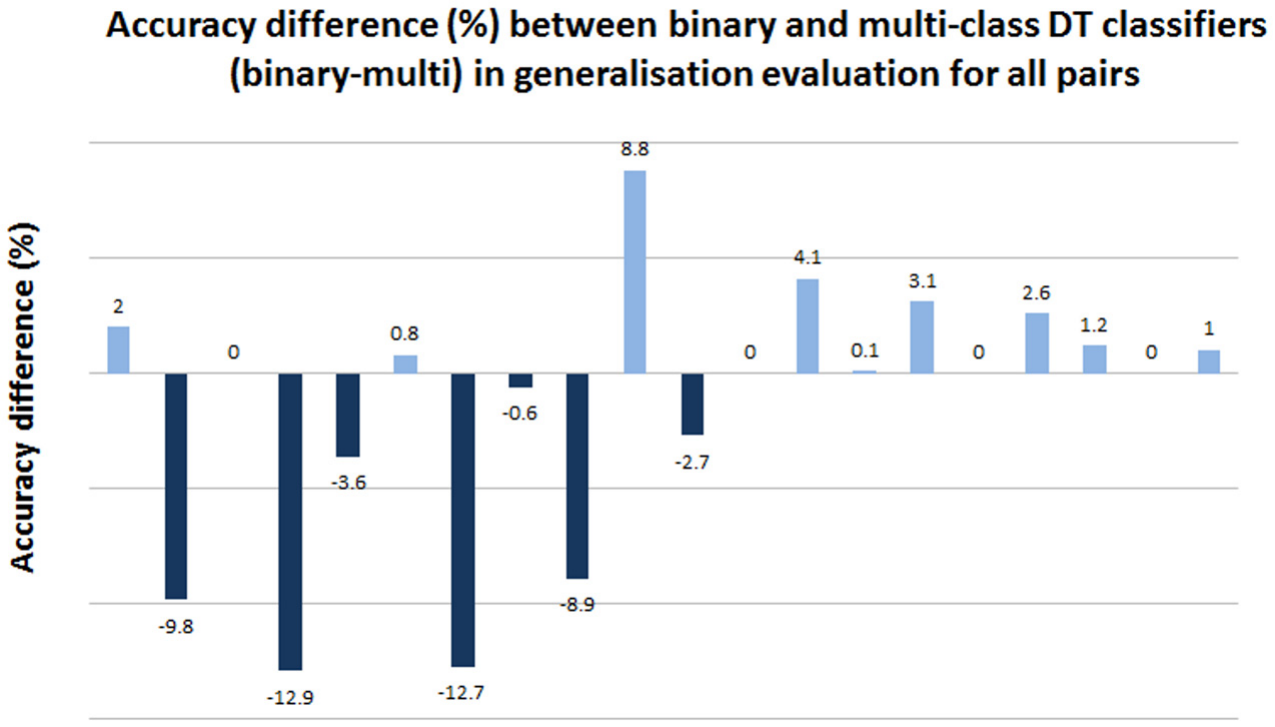
DT: Decision Tree; The 20 pairs are ordered based on the rows in Table 2; for example, the first bar is the accuracy difference applying *Caenorhabditis elegans* model to *Danio rerio*; the second bar is applying *Caenorhabditis elegans* to *Drosophila melanogaster* and so on.

Both binary and muti-class classifiers still have reasonably good performance, with over 80% accuracy in most cases. We found that multi-class achieves better performance and higher robustness. Decision tree binary models have 2 pairs below 70%, but there are no such occurrences in multi models. Multi-class models also have the highest number of pairs over 90%. We further calculated pairwise difference in accuracy, in Fig 6. It clearly shows that the multi-class classifier achieves much higher accuracy. Multi-class classifiers are better in 6 cases, and difference is much more distinct. The maximum difference is close to 13%.

### Future work and Conclusion

Supervised methods for duplicate detection in sequence databases show substantial promise. We found that features for meta-data, sequence similarity, and quality checks on alignments achieved the best results. In particular, meta-data has the potential to be used to identify and filter clearly distinct records. Comparing binary and multi-class classifiers, the multi-class approach performed strongly; it has the potential to detect difficult duplication cases and is more robust.

We plan to develop this work further in several directions. First, by improving both the efficiency and accuracy of duplicate detection procedures based on our findings in this study, by applying meta-data blocking and integrating expert review for hard cases. Second, by establishing large-scale validated benchmarks for testing duplicate detection methods. Last, by developing strategies for multi-organism duplicate detection. Our collection is already the largest available for this task, but we plan to collect duplicates from more organisms and from different curation perspectives, such as automatic curation in TrEMBL and submitter-based curation in INSDC. We have reported on single organism models. Training on multiple organisms simultaneously has the potential to make the models more robust.

## Supporting Information

S1 File Here we evaluated the method of Koh et al. (PDF)

S1 Fig Learning curve of SVM on *Escherichia coli*. (TIF)

S2 Fig Learning curve of decision trees on *Escherichia coli*. (TIF)

S1 Table Validation results on *Mus musculus*. (PDF)

S2 Table Error analysis on decision trees. (PDF)

S3 Table Results comparing binary with multi-class in terms of detecting different kinds of duplicates. (PDF)

S4 Table Generalisation results for binary classification. (PDF)

S5 Table Generalisation results for multi-class classification. (PDF)
