# Supplementary material for: Supervised Learning for Detection of Duplicates in Genomic Sequence Databases
Source: PLoS One. 2016 Aug 4;11(8):e0159644. doi: 10.1371/journal.pone.0159644 (PMC4973881; doi:10.1371/journal.pone.0159644)
Supplement: S1 File — (PDF) [file pone.0159644.s001.pdf]

We used the same feature representations and association rule mining to train one-class data as in the original published method [1]. The detailed reproduction and evaluation is for duplicates in *Homo sapiens* [1]. Here we add a study for duplicates in *Escherichia coli*. Both results demonstrate that one-class training has poor performance.

The dataset has 2048 record pairs from E. coli in total, 1024 duplicate pairs labelled based on Swiss-Prot expert curation, and another 1024 randomly selected distinct pairs.

We computed the similarity score and used the exact representations for description, literature, length, and identity. The original method contains two more features: data source and features. The current GenBank plain text format does not have data source information. Records also do not have common features in this dataset and the original method did not provide sufficient detail to reproduce these features, and hence we did not include them. The training set contains 512 duplicate pairs (one-class training in the original), and the testing set has another 512 duplicate pairs with all the 1,024 distinct pairs.

The top four rules, ordered by their support in the training set, are listed in Table 1. The results are almost as poor as what we evaluated for duplicates from *Homo sapiens*. Most of the rules are artefacts of the underlying data. For instance, the first rule is that, if a pair does not share the same reference, then they are duplicates. This does not seem to be a rule that would be robust to generalisation. The poor performance of the two evaluations on different organisms strongly supports our analysis of the method.

**Table 1. Evaluating the existing method in E. coli dataset.**

| Rule | Support | Precision (%) | Recall (%) |
|---|---|---|---|
| Sim(Literature) = 0.0 → Duplicates | 0.79 | 27.93 | 77.35 |
| Sim(Identity) = 0.9 → Duplicates | 0.66 | 58.76 | 65.64 |
| Sim(Identity) = 1.0 → Duplicates | 0.32 | 35.77 | 32.82 |
| Sim(Description) = 0.1 → Duplicates | 0.27 | 25.91 | 23.22 |

Sim stands for similarity function.

## References

1. Chen Q, Zobel J, Verspoor K. Evaluation of a Machine Learning Duplicate Detection Method for Bioinformatics Databases. ACM Ninth International Workshop on Data and Text Mining in Biomedical Informatics at CIKM. 2015;.
