# Supplementary figures and images for: Supervised Learning for Detection of Duplicates in Genomic Sequence Databases
Source: PLoS One. 2016 Aug 4;11(8):e0159644. doi: 10.1371/journal.pone.0159644 (PMC4973881; doi:10.1371/journal.pone.0159644)

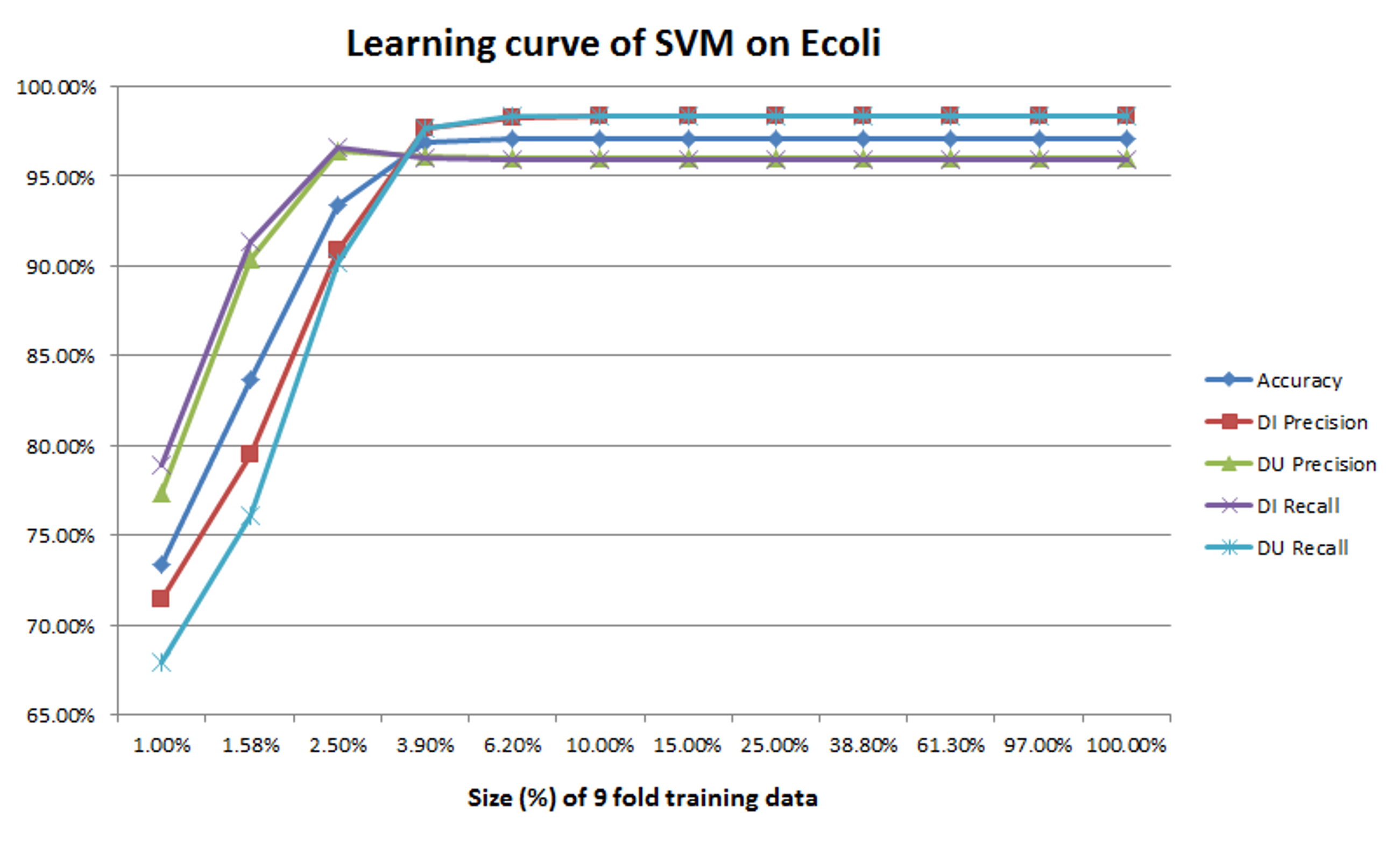

Supplement: S1 Fig — (TIF) [file pone.0159644.s002.tif]

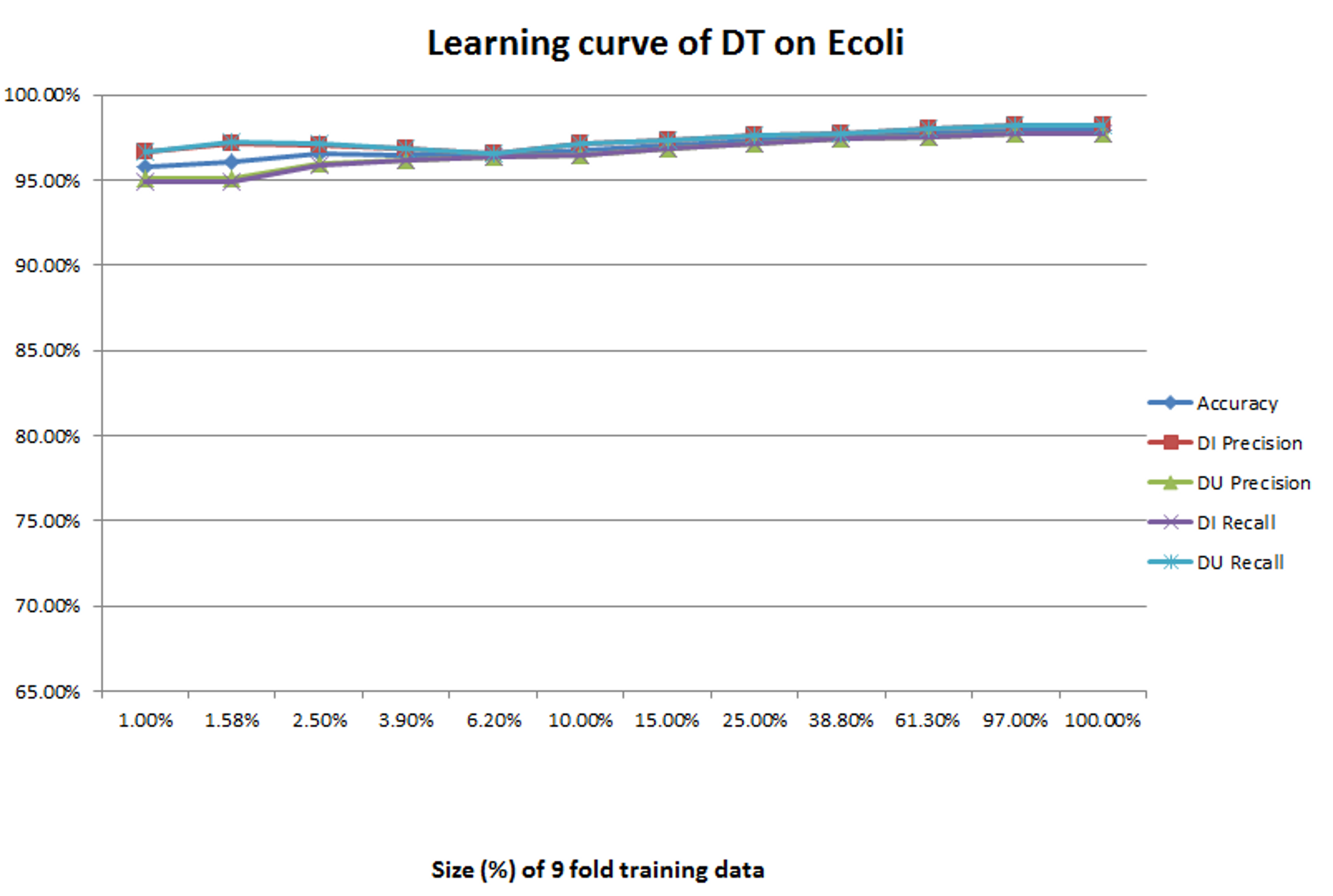

Supplement: S2 Fig — (TIF) [file pone.0159644.s003.tif]
