# Supplementary material for: Supervised Learning for Detection of Duplicates in Genomic Sequence Databases
Source: PLoS One. 2016 Aug 4;11(8):e0159644. doi: 10.1371/journal.pone.0159644 (PMC4973881; doi:10.1371/journal.pone.0159644)
Supplement: S1 Table — (PDF) [file pone.0159644.s004.pdf]

Table 1. Performance of binary classification on *Mus musculus* (AUROC=area under the receiver operator characteristic curve).

Organism	Precision		Recall		AUROC		Accuracy
	DU	DI	DU	DI	DU	DI	
Mus musculus							
Seq90	0.898	0.589	0.313	0.965	0.639	0.639	0.642
Naïve Bayes	0.984	0.859	0.836	0.987	0.951	0.951	0.912
Decision tree	**0.987**	**0.962**	**0.960**	**0.988**	**0.987**	**0.987**	**0.974**
SVM	0.957	0.949	0.948	0.959	0.953	0.953	0.953

DU: duplicate pairs; DI: distinct pairs; Accuracy is for all the instances.
