# Supplementary material for: Supervised Learning for Detection of Duplicates in Genomic Sequence Databases
Source: PLoS One. 2016 Aug 4;11(8):e0159644. doi: 10.1371/journal.pone.0159644 (PMC4973881; doi:10.1371/journal.pone.0159644)
Supplement: S2 Table — (PDF) [file pone.0159644.s005.pdf]

Table 1. Error analysis: average feature similarity for error cases on decision trees.

	Caenorhabditis		Danio rerio		Drosophila		Escherichia coli		Zea mays	
Feature	FP	FN	FP	FN	FP	FN	FP	FN	FP	FN
#Instances	98	47	215	126	532	212	14	21	20	17
Description	0.393	0.480	0.095	0.134	0.271	0.566	0.219	0.100	0.353	0.341
Literature	0.230	0.312	0.033	0.143	0.012	0.378	0.000	0.000	0.123	0.078
Length	0.306	0.497	0.094	0.091	0.390	0.714	0.471	0.317	0.449	0.467
Identity	0.863	0.897	0.912	0.978	0.910	0.934	0.938	0.981	0.918	0.960
AP	0.002	0.014	0.002	0.004	0.012	0.360	0.012	0.077	0.051	0.323
Expect_Value	0.174	0.000	0.124	0.001	0.390	0.224	0.354	0.000	0.616	0.001
CDS_Identity	0.930	0.878	0.921	0.840	0.902	0.926	0.941	0.978	0.911	0.952
CDS_AP	0.010	0.014	0.010	0.080	0.017	0.412	0.031	0.014	0.069	0.404
CDS_Expect	0.411	0.168	0.478	0.290	0.533	0.266	0.640	0.886	0.406	0.140
TRS_Identity	0.413	0.878	0.403	0.480	0.432	0.729	0.580	0.330	0.552	0.848
TRS_AP	0.020	0.062	0.020	0.097	0.027	0.494	0.039	0.022	0.113	0.512
TRS_Expect	1.322	2.528	1.522	0.767	1.807	0.938	0.921	1.623	3.056	0.044

#Instances: number of instances; FP: false positives, distinct pairs classified as duplicates; FN: false negatives, duplicates classified as distinct pairs; Numbers are averages, excluding pairs not have specific features.
