# Supplementary material for: Supervised Learning for Detection of Duplicates in Genomic Sequence Databases
Source: PLoS One. 2016 Aug 4;11(8):e0159644. doi: 10.1371/journal.pone.0159644 (PMC4973881; doi:10.1371/journal.pone.0159644)
Supplement: S3 Table — (PDF) [file pone.0159644.s006.pdf]

Table 1. Binary vs Multiple in finding different duplicate types.

Organism	EF	ES	NS
Caenorhabditis elegans			
Naïve Bayes	0.485,**0.578**	1.000,1.000	0.613,**0.671**
Decision tree	0.985,0.980	1.000,1.000	0.658,0.626
SVM	0.908,**0.932**	1.000,1.000	0.600,0.568
Danio rerio			
Naïve Bayes	0.479,**0.508**	1.000,1.000	0.146,**0.292**
Decision tree	0.958,**0.959**	1.000,1.000	0.483,0.326
SVM	0.988,**0.989**	1.000,1.000	0.146,0.011
Drosophila melanogaster			
Naïve Bayes	0.904,**0.909**	1.000,1.000	0.980,**0.985**
Decision tree	0.999,0.998	1.000,1.000	0.997,0.996
SVM	0.990,0.990	1.000,1.000	0.986,0.986
Escherichia coli			
Naïve Bayes	0.829,**0.916**	1.000,1.000	0.118,**0.235**
Decision tree	1.000,0.998	1.000,1.000	0.176,0.000
SVM	1.000,1.000	1.000,1.000	0.000,0.000
Zea mays			
Naïve Bayes	0.969,**0.980**	1.000,1.000	0.136,**0.712**
Decision tree	1.000,0.999	1.000,1.000	0.712,0.627
SVM	0.995,**0.996**	1.000,1.000	0.237,0.017

EF, ES and NS are duplicate categories in Table **??**; For each type, the first is for binary and the second is for multi-class.
