# Supplementary material for: Supervised Learning for Detection of Duplicates in Genomic Sequence Databases
Source: PLoS One. 2016 Aug 4;11(8):e0159644. doi: 10.1371/journal.pone.0159644 (PMC4973881; doi:10.1371/journal.pone.0159644)
Supplement: S4 Table — (PDF) [file pone.0159644.s007.pdf]

Table 1. Generalisation results of binary classifier.

**Organism**	Cae	Dan	Dro	Esc	Zea
Caenorhabditis elegans					
Decision tree	–	0.847	0.610	0.976	0.692
SVM	–	0.783	0.384	0.971	0.505
Danio rerio					
Decision tree	0.867	–	0.841	0.780	0.982
SVM	0.829	–	0.714	0.917	0.643
Drosophila melanogaster					
Decision tree	0.733	0.852	–	0.935	0.994
SVM	0.711	0.848	–	0.852	0.988
Escherichia coli					
Decision tree	0.845	0.764	0.986	–	0.984
SVM	0.853	0.719	0.833	–	0.966
Zea mays					
Decision tree	0.818	0.873	0.861	0.967	–
SVM	0.897	0.701	0.849	0.894	–

EF: close to or exact fragments; ES: close to or exact sequences; NS: non-significant alignments; DI refers to distinct pairs.
