# Supplementary material for: Supervised Learning for Detection of Duplicates in Genomic Sequence Databases
Source: PLoS One. 2016 Aug 4;11(8):e0159644. doi: 10.1371/journal.pone.0159644 (PMC4973881; doi:10.1371/journal.pone.0159644)
Supplement: S5 Table — (PDF) [file pone.0159644.s008.pdf]

Table 1. Generalisation results of multi-class classifier.

**Organism**	Cae	Dan	Dro	Esc	Zea
Caenorhabditis elegans					
Decision tree	–	0.827	0.708	0.976	0.821
SVM	–	0.800	0.436	0.946	0.433
Danio rerio					
Decision tree	0.903	–	0.833	0.907	0.988
SVM	0.837	–	0.725	0.963	0.668
Drosophila melanogaster					
Decision tree	0.822	0.764	–	0.962	0.994
SVM	0.711	0.741	–	0.915	0.954
Escherichia coli					
Decision tree	0.804	0.763	0.955	–	0.984
SVM	0.830	0.656	0.823	–	0.909
Zea mays					
Decision tree	0.792	0.861	0.861	0.957	–
SVM	0.863	0.741	0.842	0.895	–

EF: close to or exact fragments; ES: close to or exact sequences; NS: non-significant alignments; DI refers to distinct pairs.
